# Supplementary material for: Multiplex detection of “Candidatus Liberibacter asiaticus” and Spiroplasma citri by qPCR and droplet digital PCR
Source: PLoS One. 2021 Mar 17;16(3):e0242392. doi: 10.1371/journal.pone.0242392 (PMC7968697; doi:10.1371/journal.pone.0242392)
Supplement: S1 Table — (DOCX) [file pone.0242392.s001.docx]

**S1 Table. Quantitative data of “*Candidatus* Liberibacter asiaticus” and *Spiroplasma citri* plasmid DNA with RNR and ORF1 primers in ddPCR assays.**

| ***“Candidatus* Liberibacter asiaticus” - RNR** | | | | | ***Spiroplasma citri* – SPV1 ORF1** | | | | |
| --- | --- | --- | --- | --- | --- | --- | --- | --- | --- |
| **Calculated**  **copies/μl^a^** | **Singleplex ddPCR** | | **Duplex ddPCR** | | **Calculated**  **copies/μl^a^** | **Singleplex ddPCR** | | **Duplex ddPCR** | |
|  | **Mean** | **Poisson**  **SEM^b^** | **Mean** | **Poisson**  **SEM^b^** |  | **Mean** | **Poisson**  **SEM^b^** | **Mean** | **Poisson**  **SEM^b^** |
| 1.77E+04 | 15133.3 | 5.77 | 15480.0 | 5.77 | 1.54E+04 | 13473.3 | 5.49 | 13213.3 | 4.91 |
| 1.77E+03 | 1466.7 | 1.50 | 1534.7 | 1.53 | 1.54E+03 | 1400.0 | 1.53 | 1436.0 | 1.44 |
| 1.77E+02 | 148.7 | 0.49 | 148.0 | 0.46 | 1.54E+02 | 149.3 | 0.49 | 135.3 | 0.43 |
| 1.77E+01 | 15.2 | 0.15 | 14.5 | 0.18 | 1.54E+01 | 15.3 | 0.16 | 14.3 | 0.18 |
| 1.77E+00 | 3.1 | 0.07 | 2.3 | 0.06 | 1.54E+00 | 2.2 | 0.06 | 1.9 | 0.05 |
| NTC^c^ | 0 | 0 | 0 | 0 | NTC^c^ | 0 | 0 | 0 | 0 |

^a^Values reflect copies/20 μl ddPCR reaction. Data represents the ddPCR values from merged triplicates of each dilutions.

^b^SEM means standard error of mean.

^c^NTC means no template control.
