# Supplementary material for: Multiplex detection of “Candidatus Liberibacter asiaticus” and Spiroplasma citri by qPCR and droplet digital PCR
Source: PLoS One. 2021 Mar 17;16(3):e0242392. doi: 10.1371/journal.pone.0242392 (PMC7968697; doi:10.1371/journal.pone.0242392)
Supplement: S2 Table — (DOCX) [file pone.0242392.s002.docx]

**S2 Table. Quantitative data of “*Candidatus* Liberibacter asiaticus” and *Spiroplasma citri* dual infected citrus leaf DNA with RNR and ORF1 primers in ddPCR assays.**

| ***Candidatus* Liberibacter asiaticus - RNR** | | | | | ***Spiroplasma citri* - ORF1** | | | | |
| --- | --- | --- | --- | --- | --- | --- | --- | --- | --- |
| **Calculated**  **copies/μl^a^** | **Singleplex ddPCR** | | **Duplex ddPCR** | | **Calculated**  **copies/μl^a^** | **Singleplex ddPCR** | | **Duplex ddPCR** | |
|  | **Mean** | **Poisson**  **SEM^b^** | **Mean** | **Poisson**  **SEM^b^** |  | **Mean** | **Poisson**  **SEM^b^** | **Mean** | **Poisson**  **SEM^b^** |
| 2 ng | 75533 | 26.56 | 78133 | 27.71 | 2 ng | 16500 | 6.35 | 15200 | 5.49 |
| 0.2 ng | 7760 | 3.76 | 7533 | 3.76 | 0.2 ng | 1627 | 1.62 | 1491 | 1.50 |
| 0.02 ng | 791 | 1.10 | 790 | 1.10 | 0.02 ng | 181 | 0.55 | 141 | 0.46 |
| 0.002 ng | 69 | 0.35 | 43 | 0.29 | 0.002 ng | 19 | 0.18 | 15 | 0.19 |
| 0.0002 ng | 5 | 0.10 | 11 | 0.13 | 0.0002 ng | NA^c^ | NA^c^ | NA^c^ | NA^c^ |
| Healthy | 0 | 0 | 0 | 0 | Healthy | 0 | 0 | 0 | 0 |

^a^Values reflect copies/20 μl ddPCR reaction. Data represents the ddPCR values from merged triplicates of each dilutions.

^b^SEM means standard error of mean.

^c^NA means not applicable.
