# Supplementary material for: Multiplex detection of “Candidatus Liberibacter asiaticus” and Spiroplasma citri by qPCR and droplet digital PCR
Source: PLoS One. 2021 Mar 17;16(3):e0242392. doi: 10.1371/journal.pone.0242392 (PMC7968697; doi:10.1371/journal.pone.0242392)
Supplement: Attachment — Submitted filename: Response to Reviewers.pdf [file pone.0242392.s003.pdf]

USDA

United States Department of Agriculture

Research, Education, and Economics
Agricultural Research Service

DATE: February 24, 2020

SUBJECT: Revision PONE-D-20-34072 R1

To: Editor PLOS ONE

From: Ray Yokomi *Ray Yokomi*

This s our rebuttal letter responding (in red font) to each point raised by the reviews.

Reviewer 1

- The writing needs to be better focused on the main objectives instead of being descriptive. The research objective is stated more clearly in Abstract Page 2, Lines 29-31. To improve focus, clarity was added in the Discussion Page 13, Lines 270-271; Lines 279-282; Page 14, Lines 287-295; Lines 298-302. The major data presented, however, needs to stay as written as documentation for the efficacy and reliability of the assays which are needed if the procedures are to be used for regulatory samples.
- In discussion section, one additional paragraph could be added to explain any new parameter or technical improvement by comparison with previous researches, as a number of papers reporting sensitive detection techniques for Clas and S. citri. Although there are numerous non-PCR-based assays developed for CLas and S. citri detection, the regulatory standard is still qPCR. ddPCR was added to serve as secondary test for more precise pathogen detection, albeit, more costly and less high throughput. Some text was added in Discussion, Page 14, Lines 293-295; Lines 298-302.

Reviewer 2

- If they were used to detect in the field, do you compare the cost of droplet and qPCR? Which one is more economic? Comparative costs and time to complete ddPCR versus qPCR was added in discussion, Page 14, Lines 287-292.
- The primer of COXf was lost 3 bases as the reference, does the amplification efficiency is the same? Thank you for pointing out this disparity. It was a typo and page 6 Table 1 has been corrected.

San Joaquin Valley Agricultural Science Center/Crop Diseases, Pests and Genetics Research Unit
9611 S. Riverbend Ave., Parlier, CA 93648-9757
Voice 559.596.2990 Fax 559.596.2992 Cell 559.356.7470 E-mail ray.yokomi@usda.gov
USDA is an Equal Opportunity Employer
